# A Detailed Experimental and Theoretical Study of the Crystalline and Electronic Structure of BaHf_(1−*x*)_Zr_*x*_S_3_ Solid Solutions (0 ≤ *x* ≤ 1)

**DOI:** 10.1002/cphc.202500466

**Published:** 2025-09-14

**Authors:** Lorenza Romagnoli, Alessandro Motta, Alessandro Latini

**Affiliations:** ^1^ Dipartimento di Chimica Sapienza Università di Roma Piazzale Aldo Moro 5 00185 Roma Italy

**Keywords:** chalcogenide perovskites, density functional theory, powder X‐ray diffraction

## Abstract

Solid solutions of the chalcogenide perovskites BaHfS_3_ and BaZrS_3_ are synthesized and studied in detail across the entire composition interval by X‐ray diffraction, UV‐vis spectroscopy, and theoretical calculations. The results obtained extend those previously obtained by the same authors in previous works, giving a deeper insight into the structure‐electronic properties relationship of these materials. Furthermore, high‐resolution synchrotron radiation powder X‐ray diffraction data are obtained for pure BaHfS_3_ and BaZrS_3_. In particular, the analysis of a higher number of compositions reveals that the cell volume of the solid solutions decreases linearly with increasing Hf content, while the unit cell axes, though showing a decreasing behavior with increasing Hf content, do not show a well‐defined trend. The bandgap values of the solid solutions show a more complex relationship with the composition and unit cell volume, that is, values of Hf/(Hf + Zr) ratio below 40% do not exert a significant influence on the bandgap value, which remains practically constant, and then it increases substantially up to reach the value for pure BaHfS_3_. The comparison of the experimental data with density functional theory calculations reveals a satisfactory agreement.

## Graphical abstract

A detailed study of the structural and electronic properties of solid solutions of chalcogenide perovskites BaHf_(1−*x*)_Zr_*x*_S_3_ by means of experimental (powder X‐ray diffraction and UV‐vis spectroscopy) and theoretical calculations is presented. The study reveals that the cell parameters decrease with Hf content, while its effect on bandgap values becomes significant only for values of Hf/(Hf + Zr) ratio over 45%.

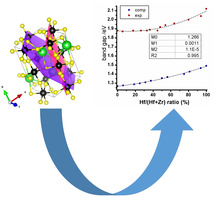

## 1 Introduction

Notwithstanding the impressive semiconductor properties of hybrid metal halide perovskites (HMHPs)^[^ ^1^ ^]^ and their excellent photovoltaic performances, with single‐junction perovskite solar cells (PSCs) having recently reached 27% power conversion efficiency (PCE) and perovskite tandem cells being already over 30%, according to NREL efficiency chart,^[^ ^2^ ^]^ the inherent instability of these materials^[^ ^3^, ^4^, ^5^, ^6^, ^7^ ^]^ and their rapid degradation under the effect of physical and chemical agents^[^ ^8^, ^9^, ^10^ ^]^ make their prospects of diffusion on a vast scale quite uncertain. The substitution of tin for lead to circumvent toxicity concerns does not appear to offer a really viable solution: besides still lagging behind lead‐containing materials in terms of PCE,^[^ ^11^ ^]^ tin halide perovskites suffer from oxidation of Sn(II)–Sn(IV)^[^ ^12^ ^]^ and show inferior thermal stability, compared to their lead‐based analogues.^[^ ^13^ ^]^

An alternative approach, first proposed in 2015,^[^ ^14^ ^]^ might be represented by the use of inorganic chalcogenide perovskites (CPs), such as BaZrS_3_ and BaHfS_3_; for this reason, research on this class of materials has received in the last few years a strong and constantly increasing boost.^[^ ^15^, ^16^, ^17^, ^18^, ^19^ ^]^ Among the advantages of CPs, there is, along with outstanding semiconductor properties (i.e., bandgap values in the useful range for photovoltaics, especially for tandem cells, and very high absorption coefficients in the visible range), an exceptional thermal and chemical stability in typical operation conditions of solar cells,^[^ ^20^ ^]^ thanks to their ceramic character. Regrettably, in contrast to the various low‐temperature, solution‐based methods commonly used for the synthesis of HMHPs, both as bulk crystals and as thin films, a limited number of strategies were known until recent years for the preparation of CPs,^[^ ^21^, ^22^, ^23^, ^24^, ^25^, ^26^ ^]^ demanding very high temperatures (700–1100 °C) and/or long reaction times, highly toxic reagents (CS_2_ or H_2_S) or, otherwise, inevitably leading to the formation of contaminating phases that are difficult to remove, and this has hitherto hindered experimental work on these materials.

Although the deposition of thin films is still challenging, notable advancements have been achieved lately,^[^ ^27^, ^28^, ^29^ ^]^ besides improved strategies for the synthesis of CPs in polycrystalline form;^[^ ^30^, ^31^ ^]^ in particular, a recently proposed solid‐state method, making use of BaS and elemental precursors (S and transition metals, namely Zr and Hf), has been employed to obtain solid solutions of general formula BaHf_(1−*x*)_Zr_*x*_S_3_ (0 ≤ *x* ≤ 1).^[^ ^32^ ^]^ This approach has shown the complete miscibility of BaZrS_3_ and BaHfS_3_ in the entire range of composition, already predicted theoretically^[^ ^33^ ^]^ and in contrast with the previously attempted Ti‐alloying of BaZrS_3_ (since BaTi_*x*_Zr_(1−*x*)_S_3_ were experimentally found to form solid solutions only for *x *< 0.1^[^ ^34^ ^]^), and the tunability of structural and optical properties of these solutions. Subsequently, simulations of the photovoltaic performances of various BaHf_(1−*x*)_Zr_*x*_S_3_ compositions have been performed, suggesting the potential for high‐efficiency solar cells enabled by the possibility to tune the bandgap with the composition.^[^ ^35^ ^]^

Herein, a more detailed description of the variation of crystalline structure and optoelectronic properties as a function of composition of BaHf_(1−*x*)_Zr_*x*_S_3_ solid solutions is reported. BaZrS_3_, BaHfS_3_, and different mixed‐metal perovskites, covering all the compositional range, were synthesized; their cell parameters were accurately determined by powder X‐ray diffraction, using both laboratory source and synchrotron radiation; and their bandgaps were measured by diffuse reflectance UV‐visible spectroscopy. Finally, the experimentally found structural and optical properties were also compared with the results of computational modeling.

## 2 Results and Discussion

### 2.1 Powder X‐Ray Diffraction

**Figure 1:**
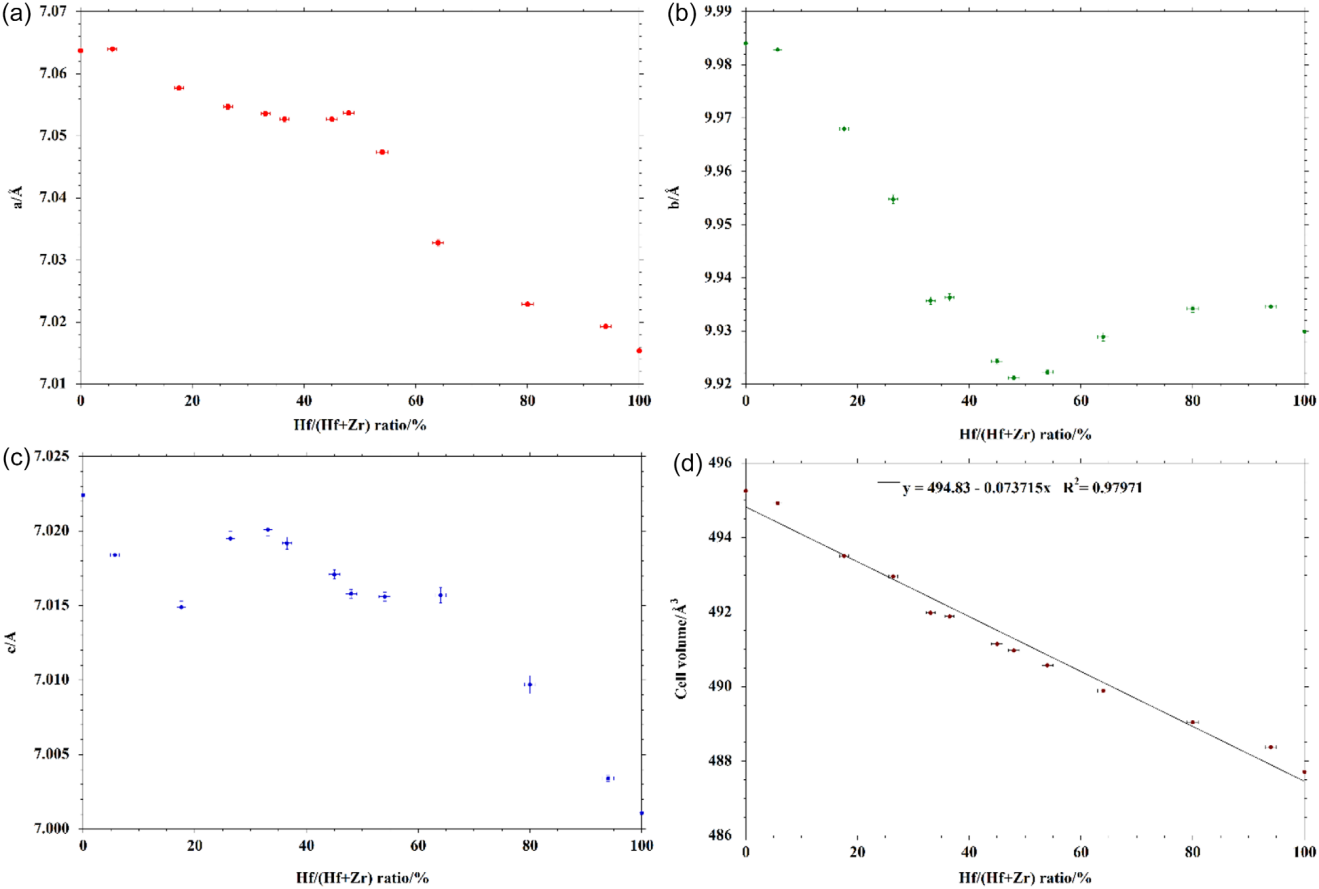
a) *a* axis length versus Hf/(Hf + Zr) ratio; b) *b* axis length versus Hf/(Hf + Zr) ratio; c) *c* axis length versus Hf/(Hf + Zr) ratio; d) unit cell volume versus Hf/(Hf + Zr) ratio.

The diffraction patterns of the samples, together with the calculated ones, their differences, and *R*_wp_ values, are reported in the Supporting Information file (Figure S1–S13, Supporting Information). In **Table** **1**, the unit cell parameters are shown, while the trends for the crystallographic axes *a*, *b*, *c*, and cell volume are shown in **Figure** **1**a–d, respectively.

**Table 1:** Crystal structure parameters of the BaHf_(1−*x*)_Zr_*x*_S_3_ compositions analyzed and their bandgap values. The uncertainties on the last digit for every parameter are given in parentheses.

Hf/(Hf + Zr) [%]	*a* [Å]	*b* [Å]	*c* [Å]	V [Å^3^]	Bandgap [eV]
100	7.01542(5)	9.92991(6)	7.00109(5)	487.713(6)	2.12(1)
94 ± 1	7.0193(2)	9.9346(3)	7.0034(2)	488.38(3)	2.04(1)
80 ± 1	7.0229(3)	9.9342(7)	7.0097(6)	489.05(3)	2.00(1)
64 ± 1	7.0328(5)	9.9289(8)	7.0157(5)	489.89(3)	1.96(1)
54 ± 1	7.0474(3)	9.9223(4)	7.0156(3)	490.58(3)	1.94(1)
48 ± 1	7.0537(3)	9.9212(4)	7.0158(3)	490.97(3)	1.92(1)
45 ± 1	7.0527(3)	9.9243(5)	7.0171(3)	491.15(3)	1.88(1)
36.5 ± 0.8	7.0527(4)	9.9363(7)	7.0192(4)	491.89(3)	1.89(1)
33.1 ± 0.8	7.0536(4)	9.9357(7)	7.0201(4)	491.99(5)	1.88(1)
26.4 ± 0.8	7.0547(4)	9.9548(8)	7.0195(5)	492.97(3)	1.88(1)
17.6 ± 0.8	7.0577(3)	9.9680(5)	7.0149(4)	493.51(3)	1.88(1)
5.7 ± 0.8	7.0640(1)	9.9829(2)	7.0184(1)	494.93(3)	1.87(1)
0	7.06374(3)	9.98406(4)	7.02243(3)	495.255(3)	1.88(1)

**Figure 2:**
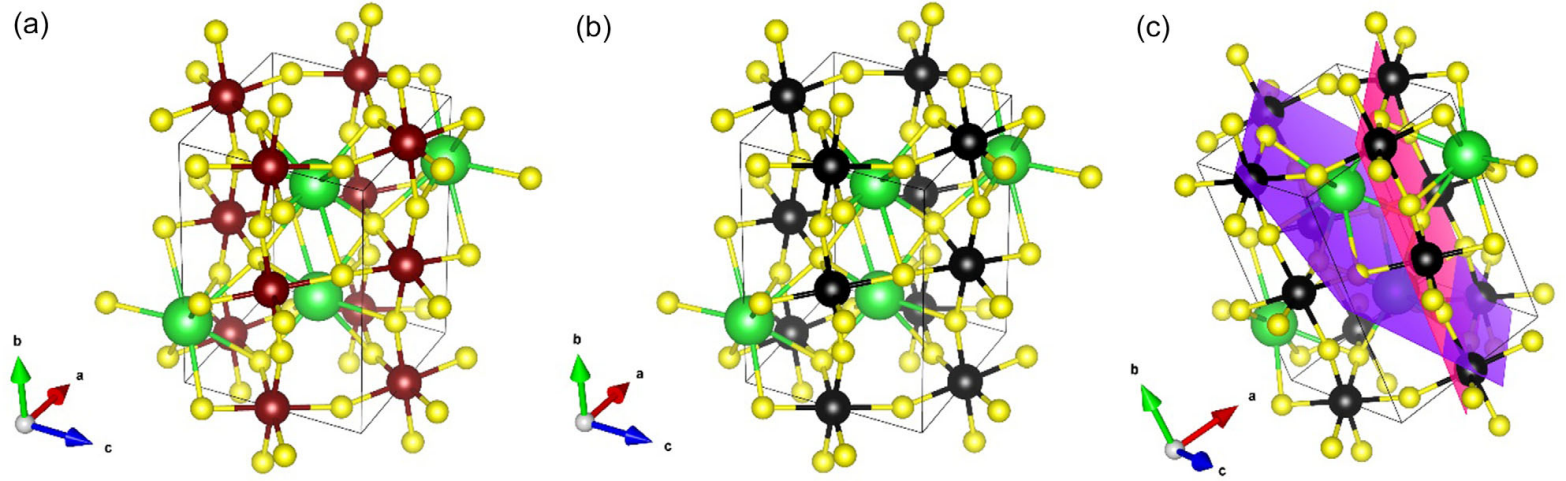
a) Crystal structure of BaHfS_3_; b) crystal structure of BaZrS_3_; c) (101) (pink) and (111) (purple) crystallographic planes in BaZrS_3_ unit cell. Yellow: sulfur; green: barium; reddish brown: hafnium; black: zirconium.

The structures were refined with the Rietveld method using the BaHfS_3_ structure and considering a random substitution of Hf atoms with the appropriate amount of Zr, obtaining good fits of the experimental patterns, with *R*_wp_ values in most cases below 8% and never reaching 10%. The random substitution between Hf and Zr agrees well with theoretical calculations (see below). Compared with the results obtained in our previous works,^[^ ^32^, ^36^ ^]^ a more detailed picture of the trends of the crystal structure parameters versus composition emerges here. While the cell axes shrink with increasing value of Hf/(Hf + Zr) ratio (molar), no very defined trend emerges, while the cell volume decreases with a very clear linear trend. The decrease of the dimensions of the unit cell with increasing Hf content can be rationalized in terms of both slightly lower ionic radius of Hf compared to Zr^[^ ^37^ ^]^ and lower electronegativity of Hf compared to Zr,^[^ ^38^ ^]^ contributing to a higher ionic character of the Hf‐S interaction and consequently to a shortening of their distances. The structures of the terminal pure phases BaHfS_3_ (**Figure** **2**a) and BaZrS_3_ (Figure 2b) agree very well with those reported in literature,^[^ ^22^, ^23^ ^]^ with only very slight differences in the positions of barium and sulfur atoms.

A very interesting trend is present in the powder patterns of the solid solutions, that is, the ratio of the background‐subtracted integrated intensities of the (101) and (111) reflections.

**Figure 3:**
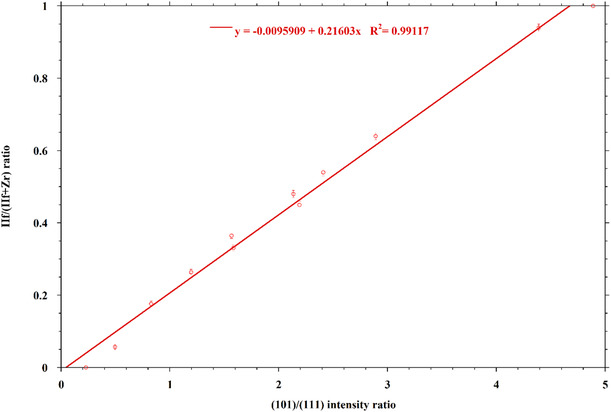
Hf/(Hf + Zr) ratio versus ratio of background‐subtracted, integrated intensities of (101) and (111) reflections.

The (101) plane reflection intensity is dominated by the effect of Hf/Zr atoms, while the (111) reflection intensity has a huge contribution from a Ba atom and a lower one of Hf/Zr atoms compared to (101) (Figure 2c). This leads to a linear increase of the (101)/(111) intensity ratio by increasing the Hf content in BaHf_(1−*x*)_Zr_*x*_S_3_, with a high value of *R* ^2^ (>0.99, **Figure** **3**), and this trend can be used as a tool to determine the composition of a solid solution, provided that no preferred orientation effect is present in the sample during the XRD pattern acquisition (Figure S14, Supporting Information). In fact, one composition point had to be discarded in the plot because it was very out of trend and, most probably, this is due to a not complete elimination of preferential orientation effects in the sample during pattern acquisition.

This method represents an alternative way to determine the composition of the BaHf_(1−*x*)_Zr_*x*_S_3_ solid solutions to that already published, which makes use of the intensity ratio of the 625 and 825 cm^−1^ peaks in the resonant Raman spectrum of the same solutions.^[^ ^36^ ^]^ Both methods are potentially very useful because of their nondestructive nature.

### 2.2 Bandgap Measurements

The bandgap values of the BaHf_(1−*x*)_Zr_*x*_S_3_ samples, obtained by performing Tauc's plots with the diffuse reflectance UV‐vis spectral data, are reported in Table 1.

**Figure 4:**
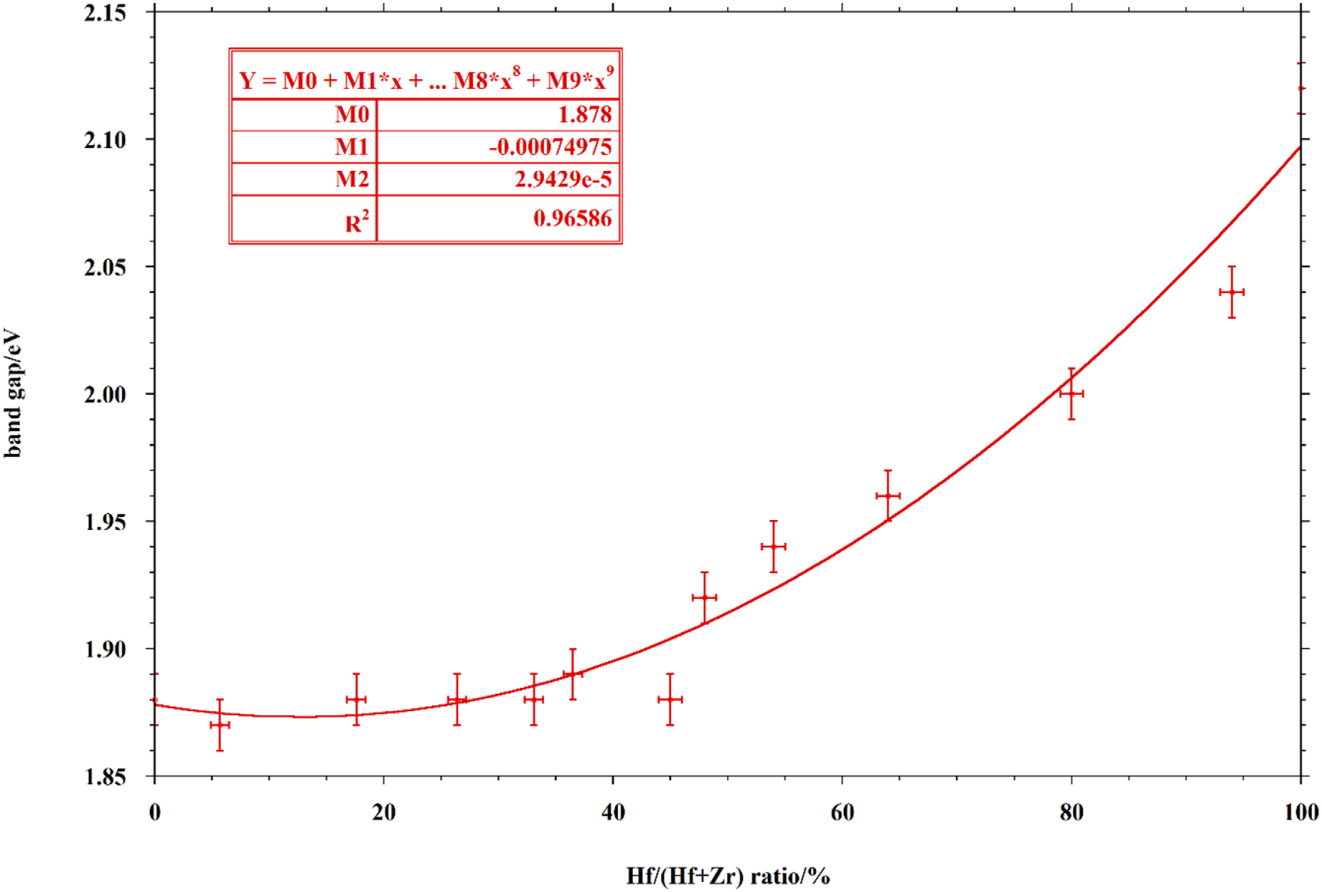
Bandgap versus Hf/(Hf + Zr) ratio.

The trend that emerges from the obtained data does not contrast with the one already found in previous work,^[^ ^32^, ^36^ ^]^ but it is more complex than it appeared with fewer samples. In fact, increasing the Hf content, up to quite high values, the observed bandgap value is practically constant (within the instrumental resolution), and then, it starts to increase for Hf/(Hf + Zr) molar ratios higher than 45% up to the value for pure BaHfS_3_. Consequently, the trend obtained for the bandgap value versus Hf/(Hf + Zr) ratio cannot be fitted satisfactorily with a linear relationship, but the use of a second‐degree equation gives a very satisfactory fit, as shown in **Figure** **4**.

In an analogous way, a parabolic fit of the bandgap value versus the unit cell volume with a very similar *R* ^2^ value can be obtained, considering the excellent linear relationship between unit cell volume and Hf/(Hf + Zr) ratio (Figure S15, Supporting Information). The present work confirms the possibility of a fine tuning of the bandgap for practical purposes by simply varying the relative concentrations of Hf and Zr in the crystal structure.

### 2.3 Computational Analysis

Structures containing both Hf and Zr ions were simulated starting from different possible arrangements of the two ions within the crystal lattice. Specifically, for each system, five configurations were considered: one with a homogeneous distribution of the two ions; one with clustered distribution where ions of the same type are grouped together; three distributions where ions are primarily arranged along the (100), (010), and (001) planes. Below, the trends in cell parameters as a function of composition are shown, comparing calculated and experimental results.

**Figure 5:**
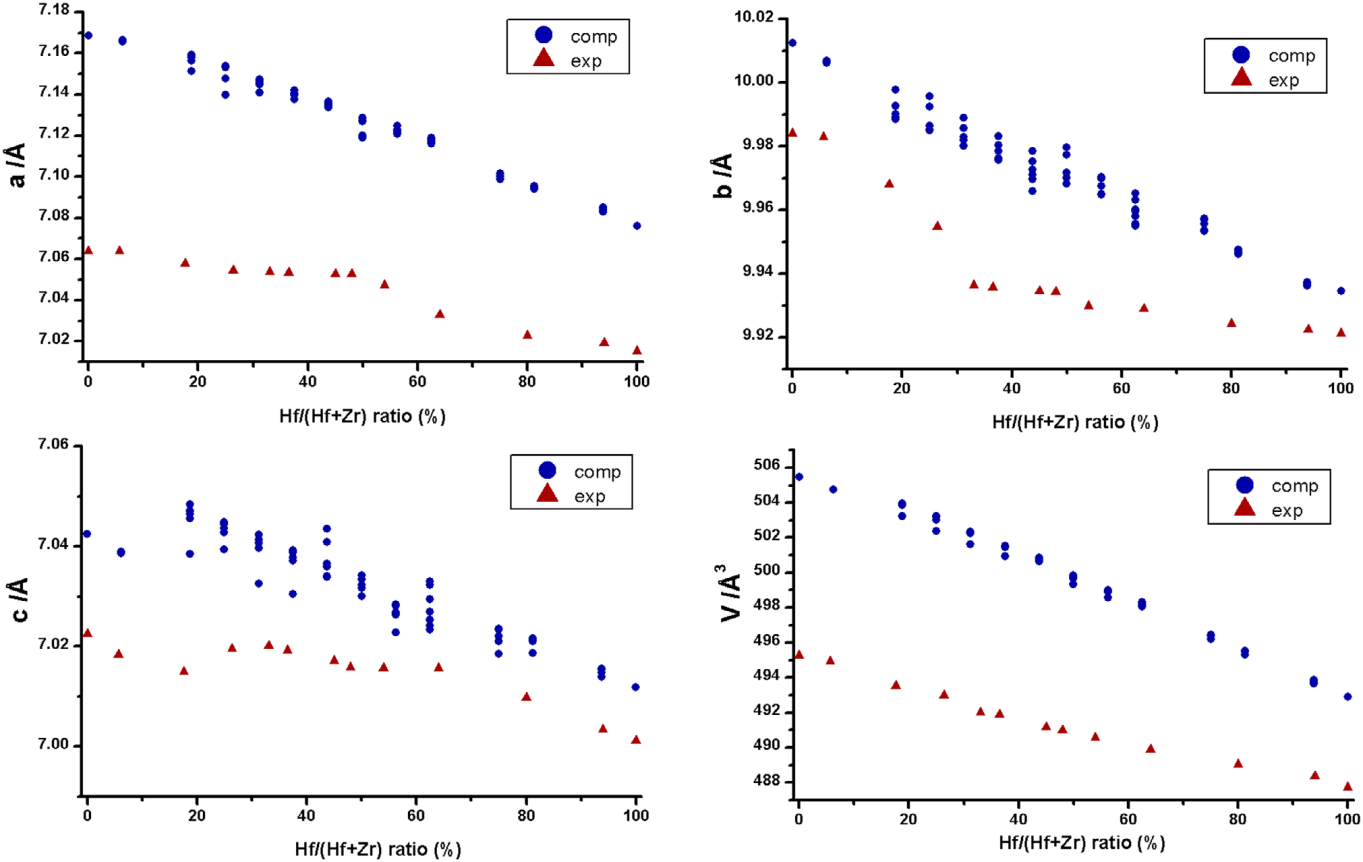
Comparison between computed and experimental values relative to the discussed cell parameters.

Regarding the three lattice parameters (**Figure** **5**), the simulated structure appears to exhibit a similar variability to that observed in the experimental results. In all cases, the calculated values are overestimated by ≈1–1.5% relative to experimental data. For the cell volume, the trends between experimental and calculated values are highly comparable. Here, both experimental and computational data show reduced variability in volume trend as a function of composition. As expected, the computed volume is also overestimated by ≈2% compared to experiments.

The linear fit performed on the trend of computed volume as a function of composition shows an intercept of 506 Å and a slope of −0.1287, in excellent agreement with the experimental results. Indeed, a deviation of 2.3% is observed for the intercept, and the slope differs by 0.05 compared to the experimental parameters.

**Figure 6:**
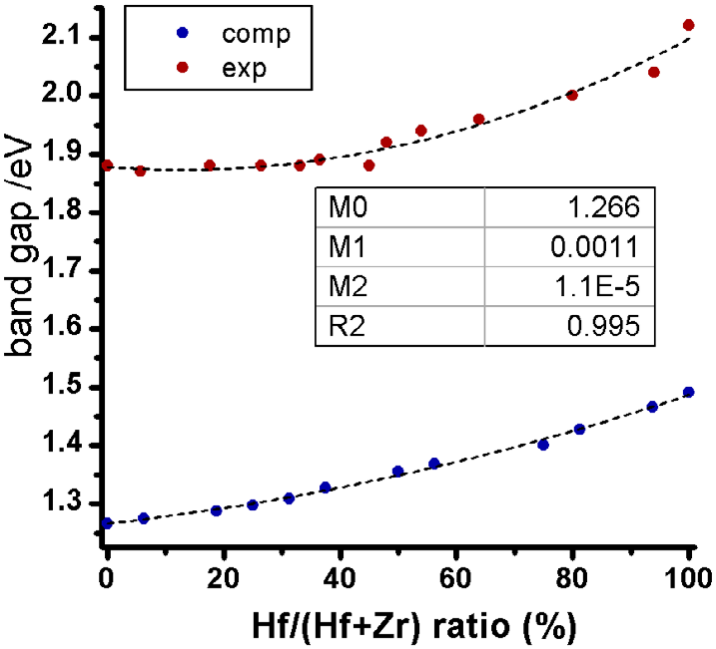
Comparison between computed and experimental bandgap versus Hf/(Hf + Zr) ratio. In the inset, the parameters obtained from the polynomial fit of the computed values. The figure displays values associated with systems where hafnium atoms are uniformly distributed within the unit cell.

The trend of the bandgap as a function of composition is shown in **Figure** **6**.

It can be observed that the computed bandgaps are always underestimated compared to the experimental values, as expected from the theoretical approach,^[^ ^39^ ^]^ but the computed and experimental trends are very similar. In fact, as the Hf percentage increases, the computed bandgap progressively widens. The polynomial fit parameters evaluated on the computed values (see inset of Figure 6) match well the experimental ones. Moreover, the attempt to perform a linear fit leads to worse R‐squared.

## 3 Conclusions

In this work, a thorough investigation of the structural and electronic properties of solid solutions between BaHfS_3_ and BaZrS_3_, covering the entire compositional range, was performed by combining powder X‐ray diffraction and diffuse reflectance UV‐vis spectroscopy experiments with state‐of‐the‐art density functional theory (DFT) calculations. A linear decrease in cell volume of the solid solutions was experimentally found to occur with increasing Hf content, while the trend for single cell parameters shows more complex behavior. The ratio of the background‐subtracted integrated intensities of (101) and (111) reflections as a function of the Hf/(Hf + Zr) ratio showed, quite surprisingly, a clearly linear trend, given the different atom contribution to those planes, which suggests this as a powerful tool for the Hf quantification in a solid solution, alternative to the use of resonant Raman spectra. As regards the dependence of the bandgap on the Hf/(Hf + Zr) ratio, the previous finding (obtained by using a lower number of solid solutions) of a linear increase of the gap with increasing Hf content reveals, with a larger number of compositions, covering all the compositional range, a better agreement with a parabolic fit. Finally, simulated structures reproduce satisfactorily the experimentally observed trends for separate cell parameters and very well the contraction in cell volume at higher values of Hf/(Hf + Zr) ratio. Also, the trend for the calculated bandgaps as a function of composition, despite the underestimation due to the theoretical approach, shows very good agreement with the experimentally measured bandgaps.

## 4 Experimental Section

4.1

4.1.1

##### Synthesis

All the reagents were used as received without any purification procedure. Barium sulfide powder (99.9%), sulfur powder (99.98% purity), and Wheaton 10 mL prescored clear borosilicate glass ampules were purchased from Merck. Zirconium powder −60 + 100 mesh (99.8% purity) was purchased from Chempur. Hafnium crystal bar (99.9% purity) was purchased from Smart‐elements GmbH. Hafnium powder (−60 + 100 mesh) was prepared by filing the crystal bar and sieving the resulting powder with meshes of appropriate sizes. The synthetic procedure (in borosilicate glass ampules flame‐sealed under vacuum, 3 × 10^−1^ mbar) and the purification of the obtained materials are the same used in previous works.^[^ ^32^, ^36^ ^]^ The particle size of Zr and Hf is extremely important, because if a too fine powder is used (e.g., 325 mesh), the synthesis becomes explosive, while with a too coarse powder the reaction is incomplete. The particle size used in this work proved to be the best compromise. No commercial supplier of hafnium powder of the appropriate particle size was found; consequently, we opted for preparing it ourselves by filing a hafnium crystal bar.

##### EDS Analysis

The composition of each sample was determined with EDS analysis by using a Thermo Fisher Phenom ProX.

##### Synchrotron Radiation Powder X‐Ray Diffraction

High‐resolution synchrotron powder X‐ray diffraction measurements^[^ ^40^, ^41^ ^]^ of pure BaHfS_3_ and BaZrS_3_ samples were performed at beamline ID31 at the European Synchrotron Radiation Facility (ESRF). The sample powders were loaded into cylindrical slots (≈1 mm thickness) held between Kapton windows in a high‐throughput sample holder. Each sample was measured in transmission geometry with an incident X‐ray energy of 75.051 keV (*λ* = 0.16520 Å). Measured intensities were collected using a Pilatus CdTe 2M detector (1679 × 1475 pixels, 172 × 172 μm^−2^ each) positioned with the incident beam in the corner of the detector. The sample‐to‐detector distance was ≈1.5 m. Background measurements for the empty windows were measured and subtracted. NIST SRM 660b (LaB_6_) was used for geometry calibration performed with the software pyFAI followed by image integration including a flat‐field, geometry, solid‐angle, and polarization corrections.

##### Powder X‐Ray Diffraction

Powder X‐ray diffraction patterns of the BaHf_(1−*x*)_Zr_*x*_S_3_ solid solutions were acquired by using a Malvern Panalytical X’Pert Pro diffractometer (Cu K*α* radiation) equipped with a capillary spinner, a graded multilayer elliptical mirror, and an ultrafast RTMS X’Celerator detector. Powdered samples were fixed on the outer surface of a 0.3 mm borosilicate glass capillary by sprinkling the powder on the capillary previously covered with a thin layer of Molykote 4 silicone grease. This procedure was used in order to minimize the background caused by the glass in the diffraction pattern, considering that the samples are highly absorbing because they contain heavy elements. A LaB_6_ diffraction pattern was also acquired in the same instrumental configuration to obtain the instrumental function in the Rietveld analysis procedures.

##### Rietveld Refinement

Both synchrotron and lab source diffraction patterns were analyzed by the Rietveld method using the MAUD (Materials Analysis Using Diffraction) software.^[^ ^42^ ^]^ Structures were refined using literature data for BaHfS_3_ and BaZrS_3_;^[^ ^22^, ^23^ ^]^ in the case of the solid solutions, the BaHfS_3_ structure was modified by inserting the appropriate amount of Zr partial occupancy. Instrumental functions were calculated for both synchrotron and lab source data using LaB_6_ diffraction patterns. The ionic radius values used (in Å) are 1.42, 0.71, 0.72, 1.84 for Ba^2+^, Hf^4+^, Zr^4+^, and S^2−^, respectively.

##### Bandgap Measurements

The bandgap of each sample was determined by using the Tauc method^[^ ^43^, ^44^ ^]^ applied on UV‐Vis diffuse reflectance spectra. The spectra were acquired in the range 220–1400 nm (resolution 0.2 nm) with a Shimadzu UV‐2600 spectrophotometer equipped with an ISR 2600 Plus integrating sphere, with BaSO_4_ as reflectance standard. The Tauc plots were obtained assuming an allowed direct bandgap transition.

##### Computational Methods

DFT‐based simulations were performed with the CP2K/Quickstep package, using a hybrid Gaussian and plane wave method.^[^ ^45^ ^]^ A double quality DZVP Gaussian basis set was employed for all the atoms.^[^ ^46^ ^]^ The Goedecker–Teter–Hutter pseudopotentials^[^ ^47^ ^]^ together with a 400 Ry plane wave cutoff were used to expand the densities obtained with the Perdew–Burke–Ernzerhof (PBE)^[^ ^48^ ^]^ exchange‐correlation density functional, and vdW forces are taken into account with the Grimme D3 Method.^[^ ^49^ ^]^ Only the gamma point was considered in a supercell approach. Periodic boundary conditions are applied in all directions of space.

The cells were optimized starting from experimental data and considering a 2 × 2 × 2 supercell, to allow for more sensitive variation of the Hf% composition. The bandgap was evaluated on the most representative optimized cells, using the Quantum ESPRESSO^[^ ^50^ ^]^ package on a uniform (3 × 2 × 3) Monkhorst–Pack grid.

## Supporting Information

Experimental versus X‐ray diffraction patterns (Figure S1–S13, Supporting Information); relative variation of the (101) and (111) reflections intensities as a function of Hf content in some BaHf_(1−*x*)_Zr_*x*_S_3_ solid solutions (Figure S14, Supporting Information); bandgap value versus unit cell volume for BaHf_(1−*x*)_Zr_*x*_S_3_ solid solutions (Figure S15, Supporting Information).

Deposition Numbers 2484374–2484386 contain the supplementary crystallographic data for this paper. These data are provided free of charge by the joint Cambridge Crystallographic Data Centre and Fachinformationszentrum Karlsruhe http://www.ccdc.cam.ac.uk/structures, Access Structures service.

## Conflict of Interest

The authors declare no conflict of interest.

## Supporting information

Supplementary Material

## Data Availability

The data that support the findings of this study are available from the corresponding author upon reasonable request.
