# Supplementary material for: A Detailed Experimental and Theoretical Study of the Crystalline and Electronic Structure of BaHf(1−x)Zr x S3 Solid Solutions (0 ≤ x ≤ 1)
Source: Chemphyschem. 2025 Sep 14;26(22):e202500466. doi: 10.1002/cphc.202500466 (PMC12640665; doi:10.1002/cphc.202500466)
Supplement: Supplementary file 1 — Supplementary Material [file CPHC-26-e202500466-s001.pdf]

Supporting Information

A Detailed Experimental and Theoretical Study of the Crystalline and Electronic Structure of $BaHf_{(1-x)}Zr_xS_3$ Solid Solutions ($0 \leq x \leq 1$)

Lorenza Romagnoli[1], Alessandro Motta[1] and Alessandro Latini*[1]*

[1]Dipartimento di Chimica, Sapienza Università di Roma, Piazzale Aldo Moro, 5 00185 Roma

**Corresponding authors*

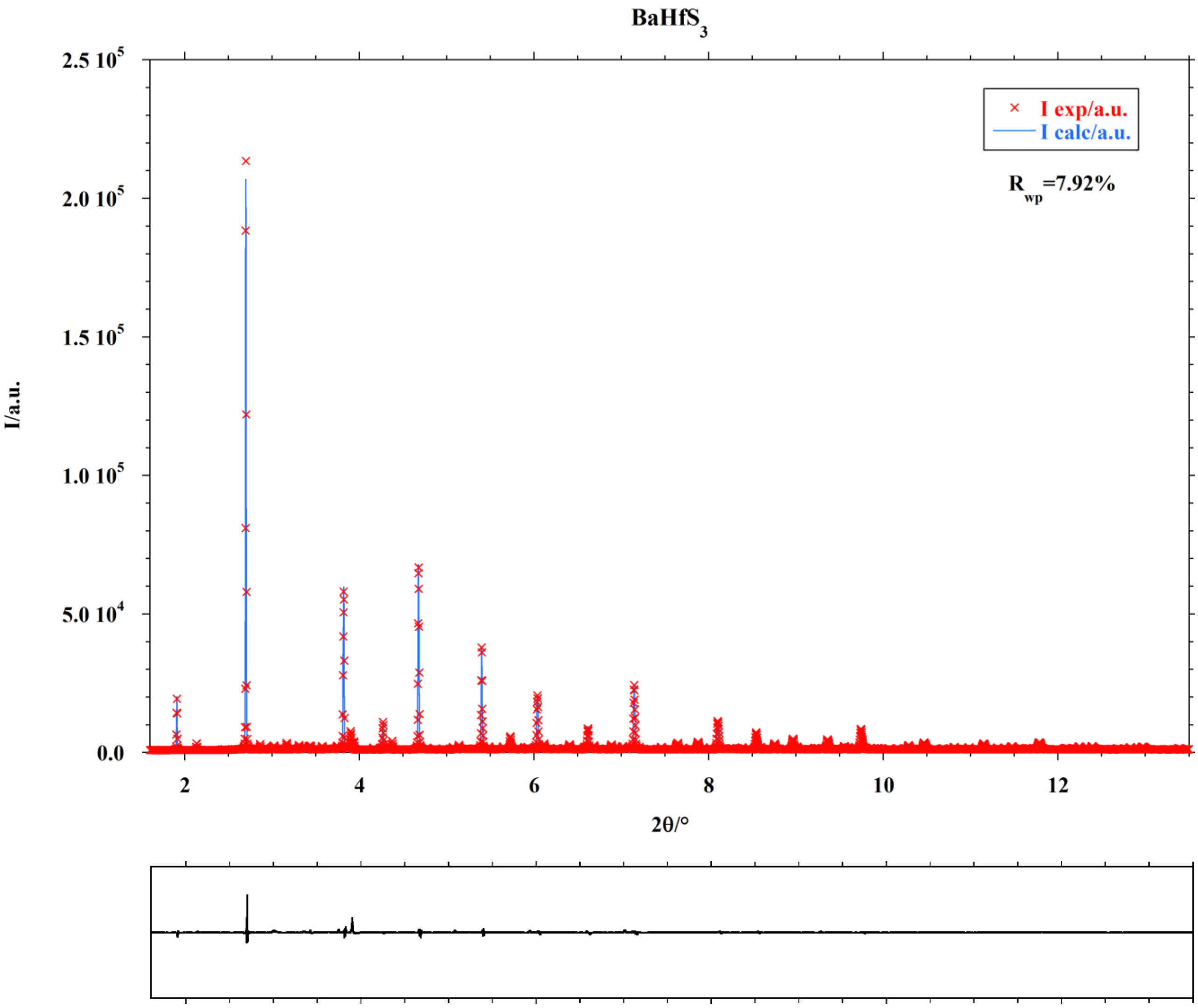

Figure S1. Experimental-calculated diffraction patterns of $BaHfS_3$ (upper panel) and difference plot (lower panel).

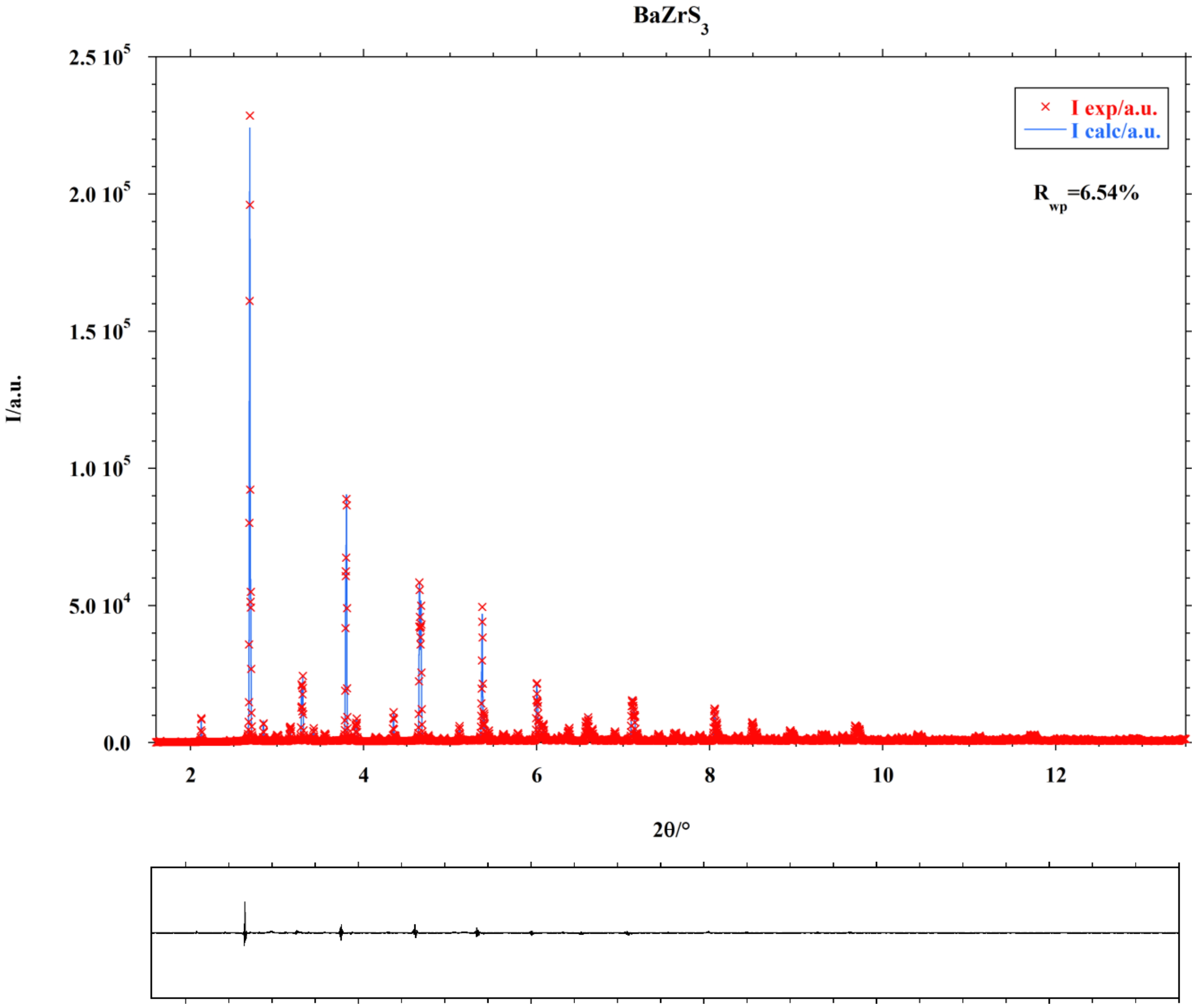

Figure S2. Experimental-calculated diffraction patterns of $BaZrS_3$ (upper panel) and difference plot (lower panel).

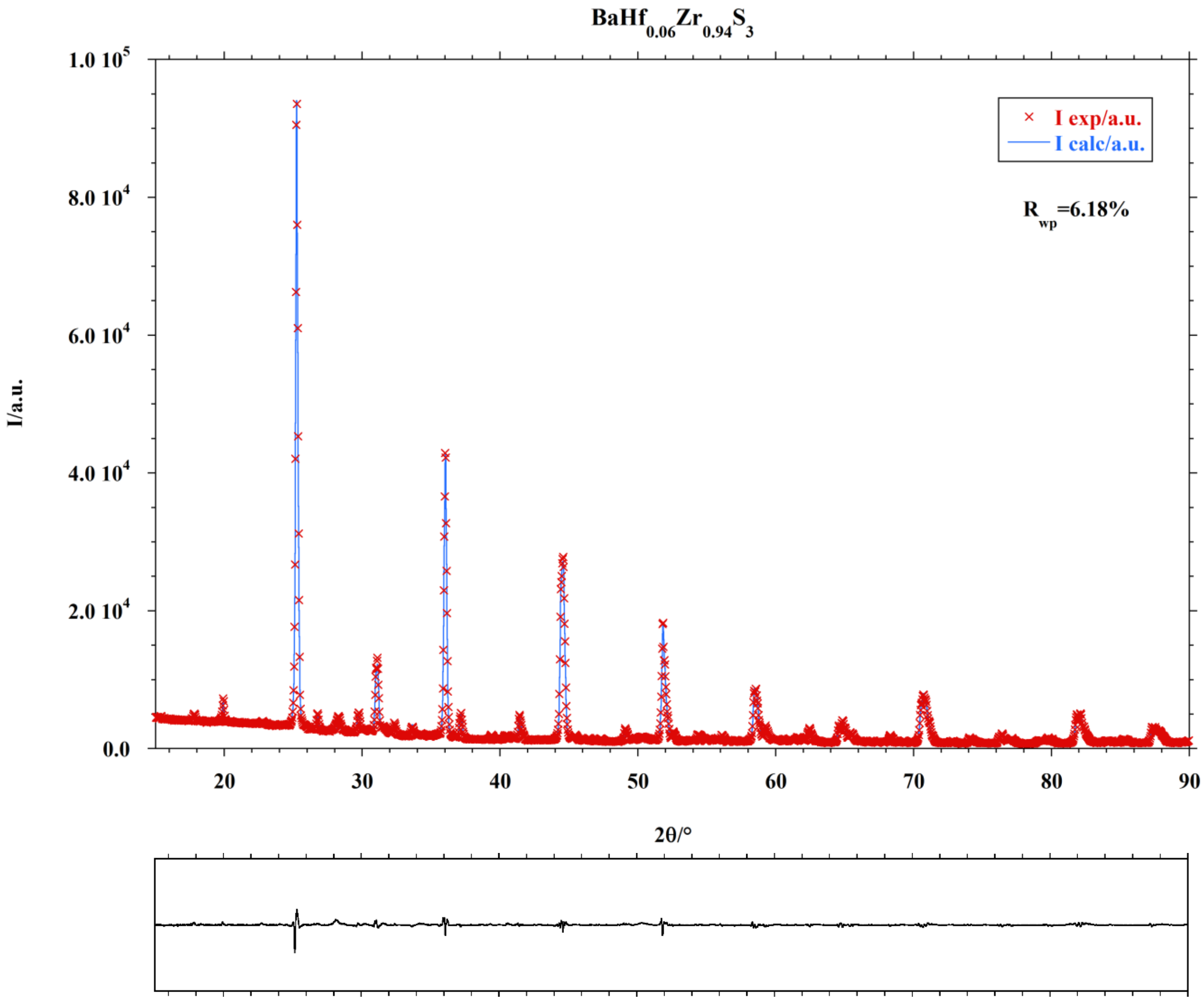

Figure S3. Experimental-calculated diffraction patterns of $BaHf_{0.06}Zr_{0.94}S_3$ (upper panel) and difference plot (lower panel).

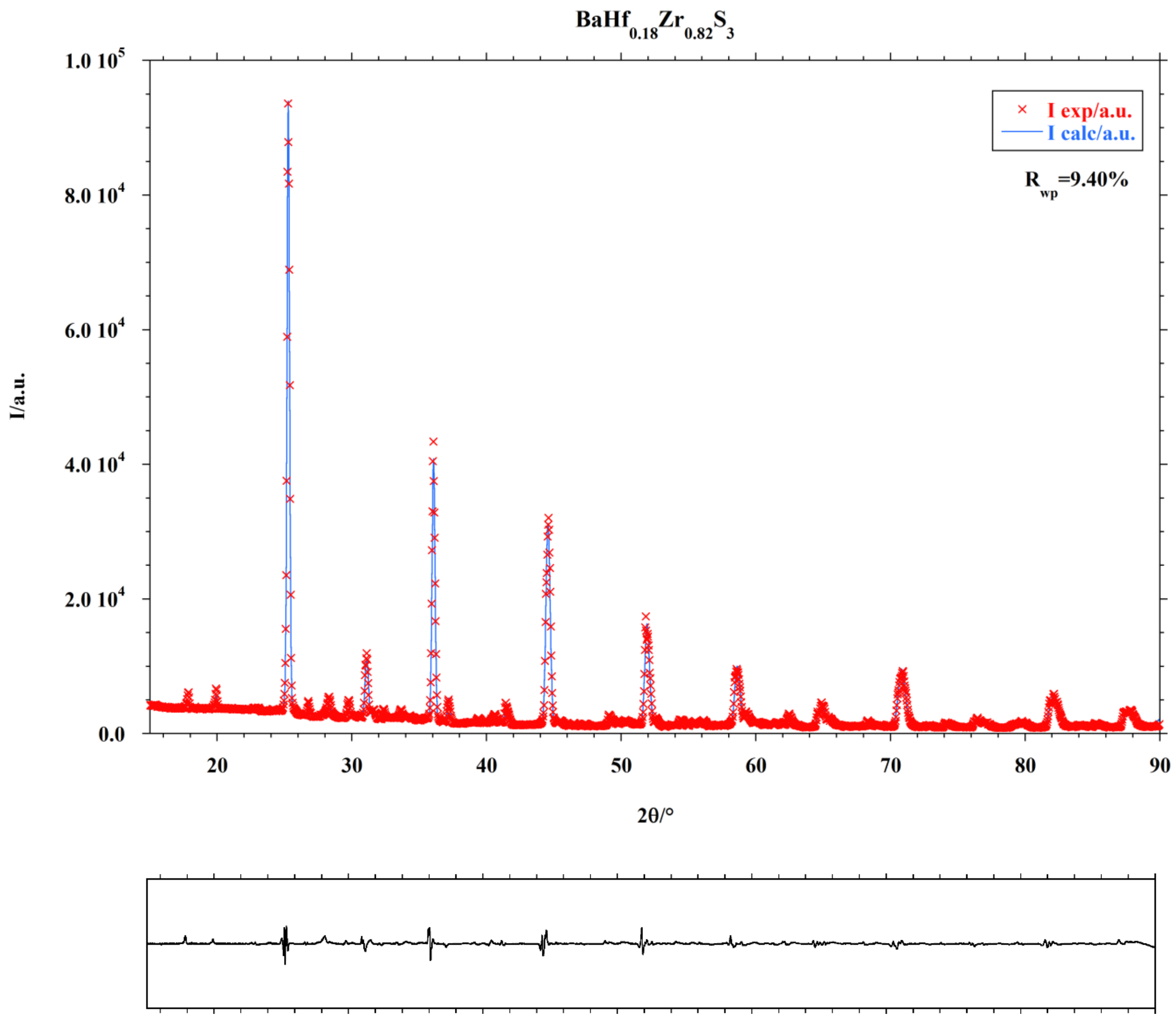

Figure S4. Experimental-calculated diffraction patterns of $BaHf_{0.18}Zr_{0.82}S_3$ (upper panel) and difference plot (lower panel).

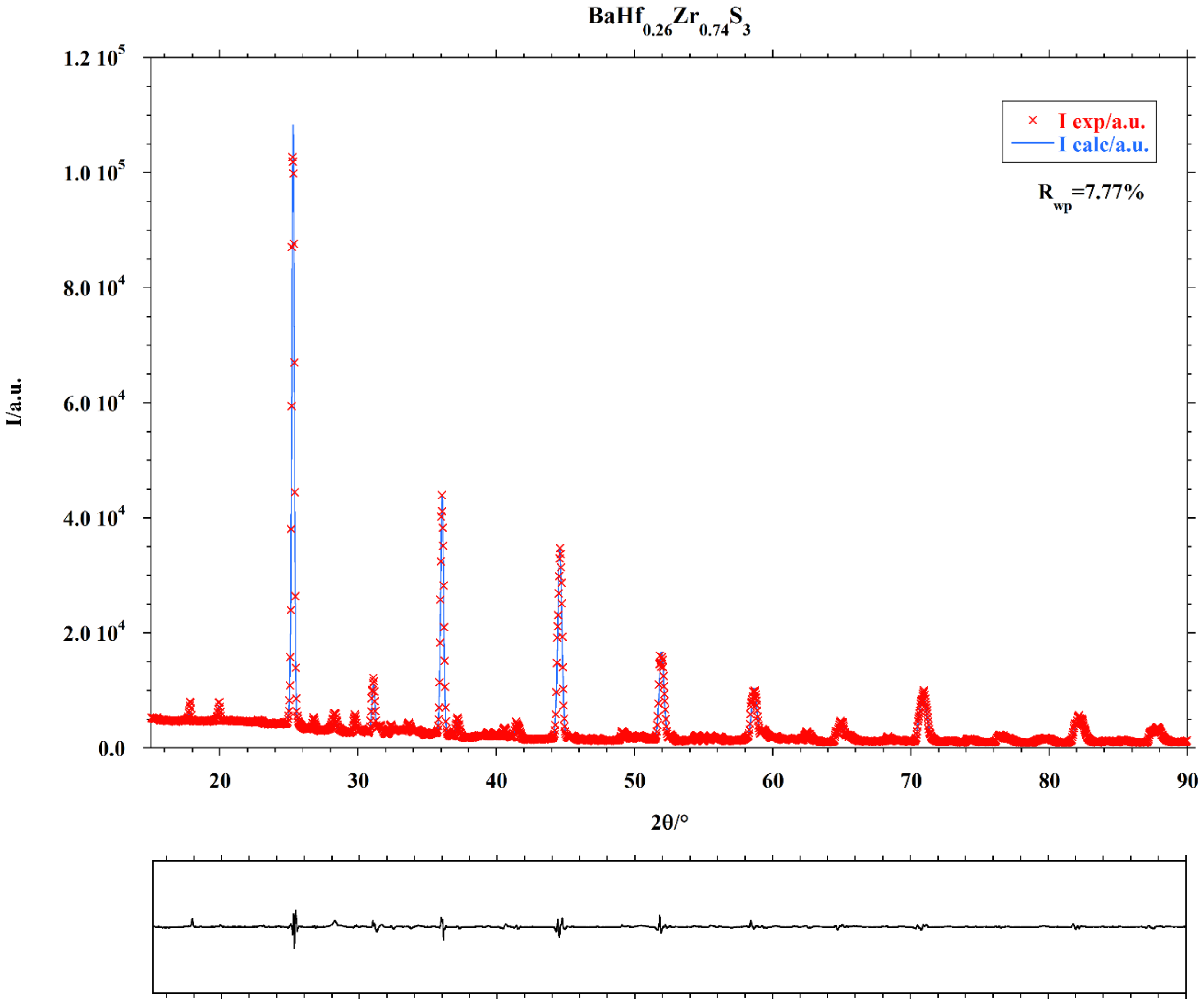

Figure S5. Experimental-calculated diffraction patterns of $BaHf_{0.26}Zr_{0.74}S_3$ (upper panel) and difference plot (lower panel).

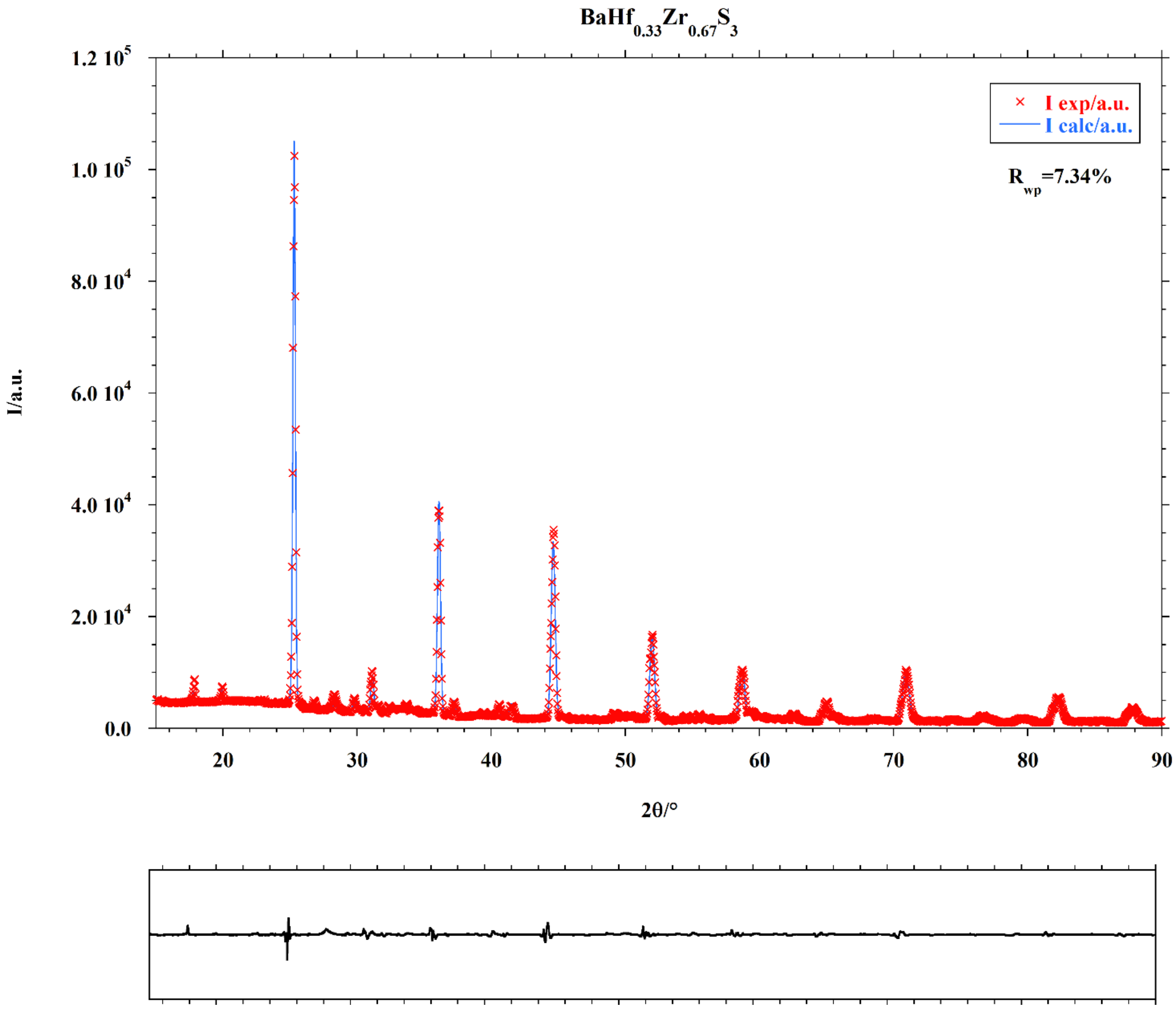

Figure S6. Experimental-calculated diffraction patterns of $BaHf_{0.33}Zr_{0.67}S_3$ (upper panel) and difference plot (lower panel).

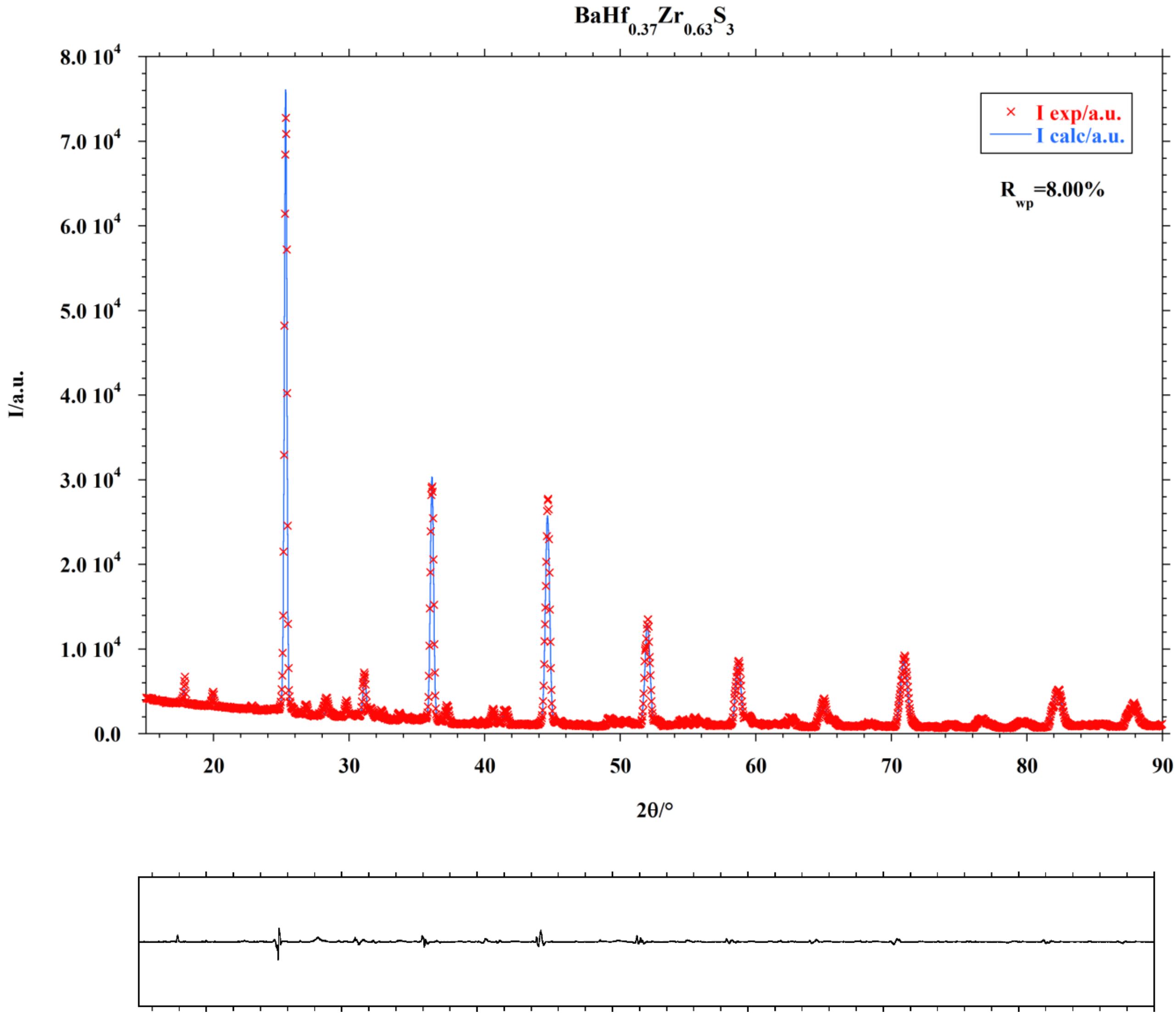

Figure S7. Experimental-calculated diffraction patterns of $BaHf_{0.37}Zr_{0.63}S_3$ (upper panel) and difference plot (lower panel).

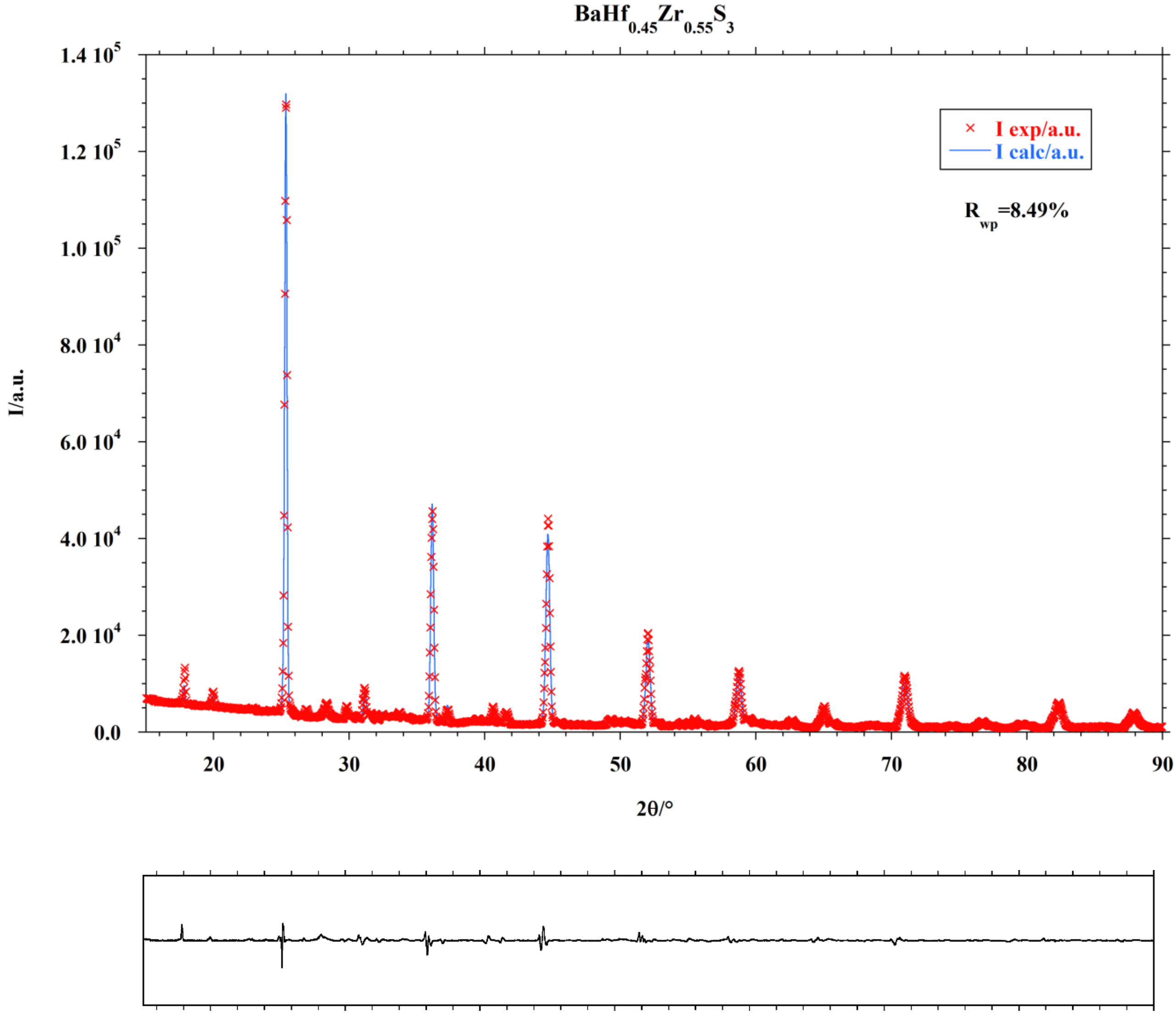

Figure S8. Experimental-calculated diffraction patterns of $BaHf_{0.45}Zr_{0.55}S_3$ (upper panel) and difference plot (lower panel).

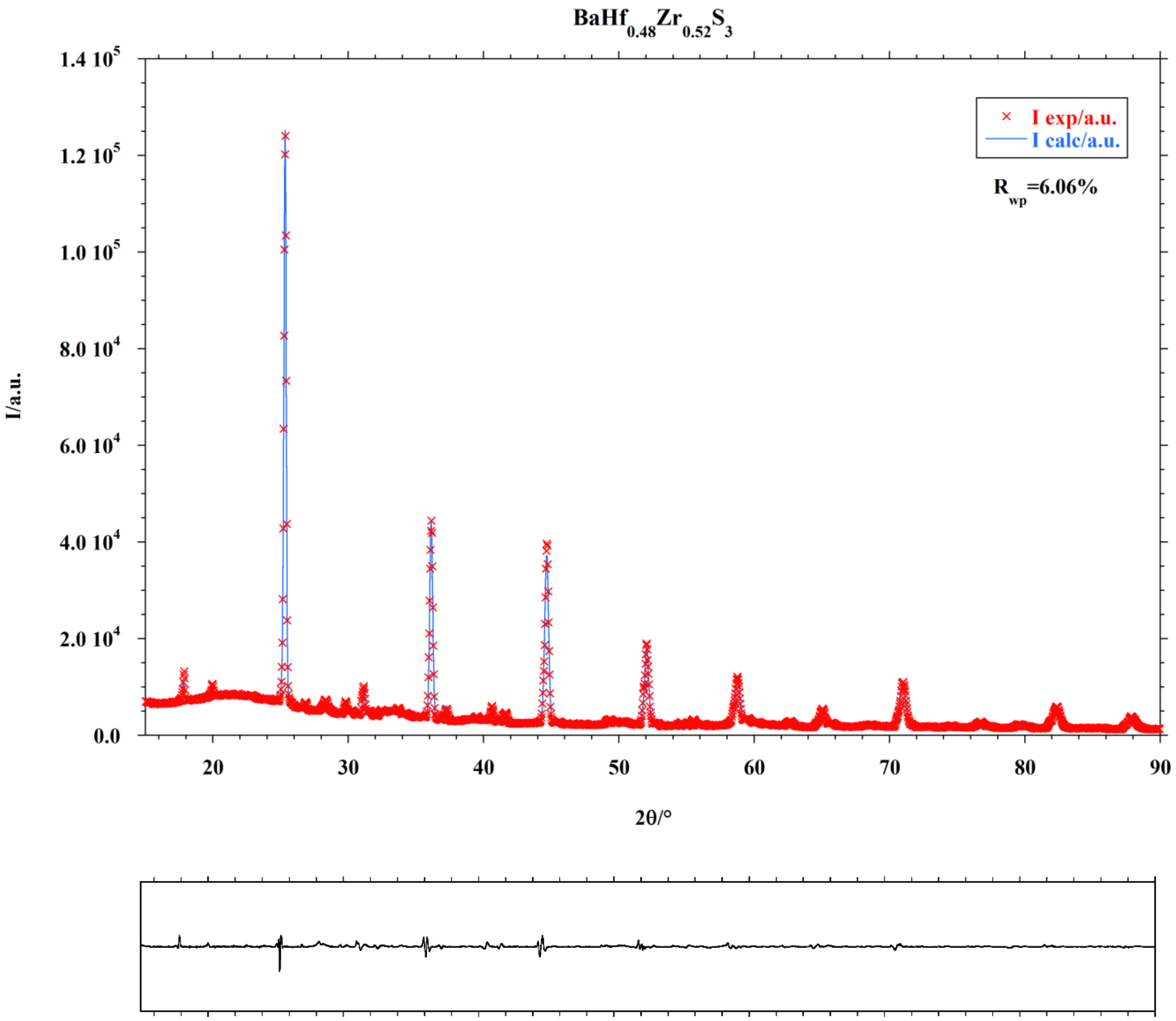

Figure S9. Experimental-calculated diffraction patterns of $BaHf_{0.48}Zr_{0.52}S_3$ (upper panel) and difference plot (lower panel).

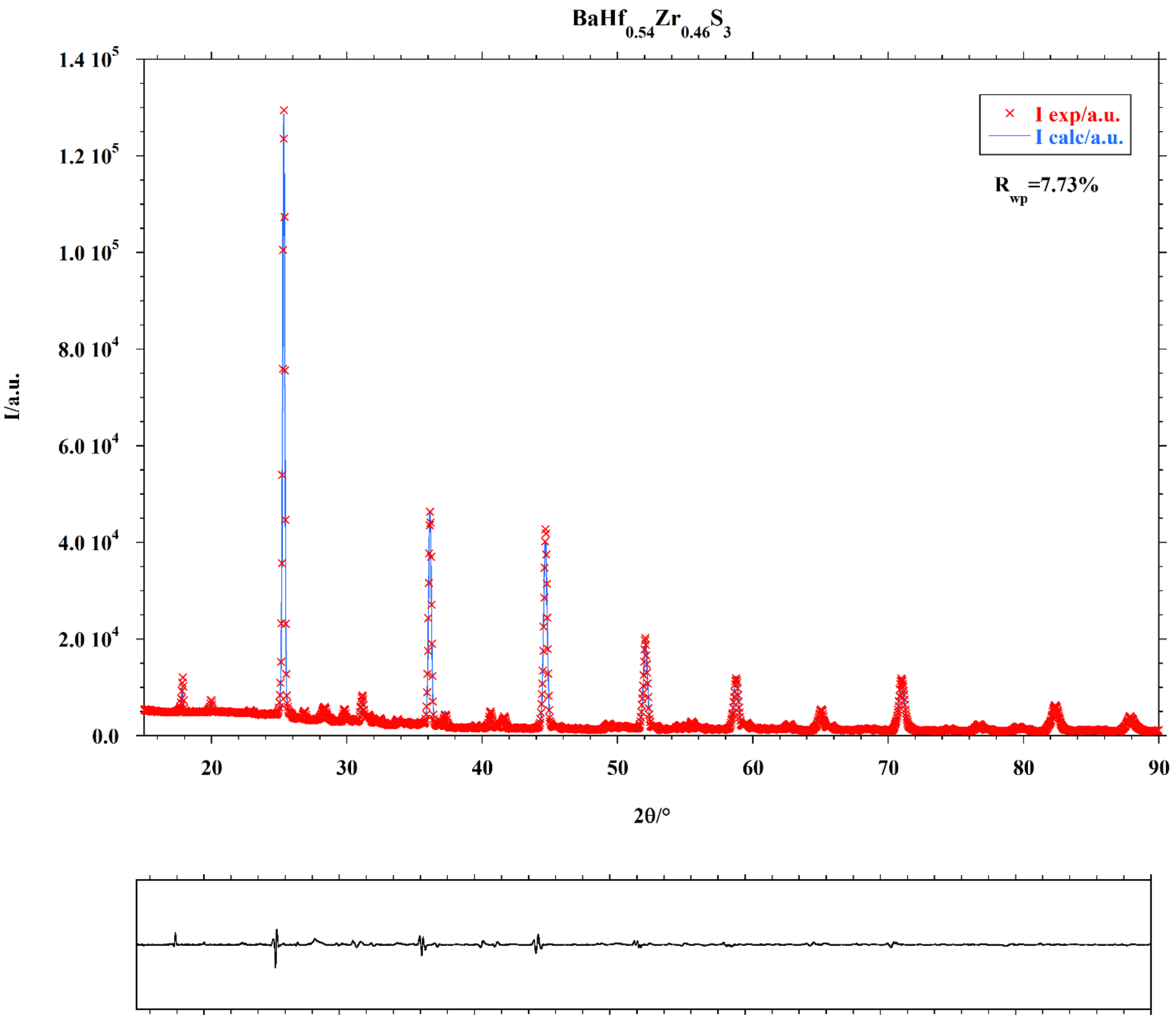

Figure S10. Experimental-calculated diffraction patterns of $BaHf_{0.54}Zr_{0.46}S_3$ (upper panel) and difference plot (lower panel).

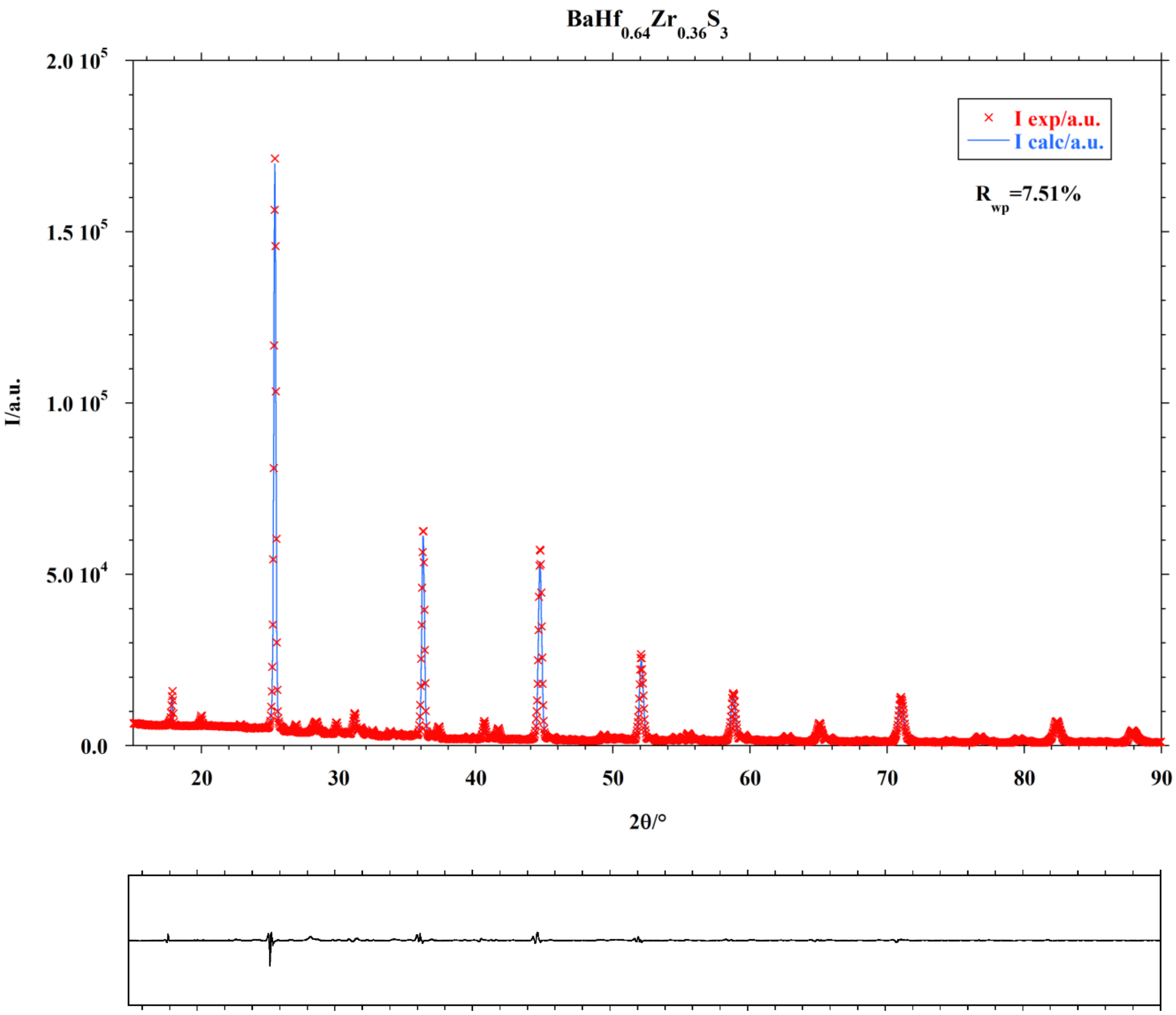

Figure S11. Experimental-calculated diffraction patterns of $BaHf_{0.64}Zr_{0.36}S_3$ (upper panel) and difference plot (lower panel).

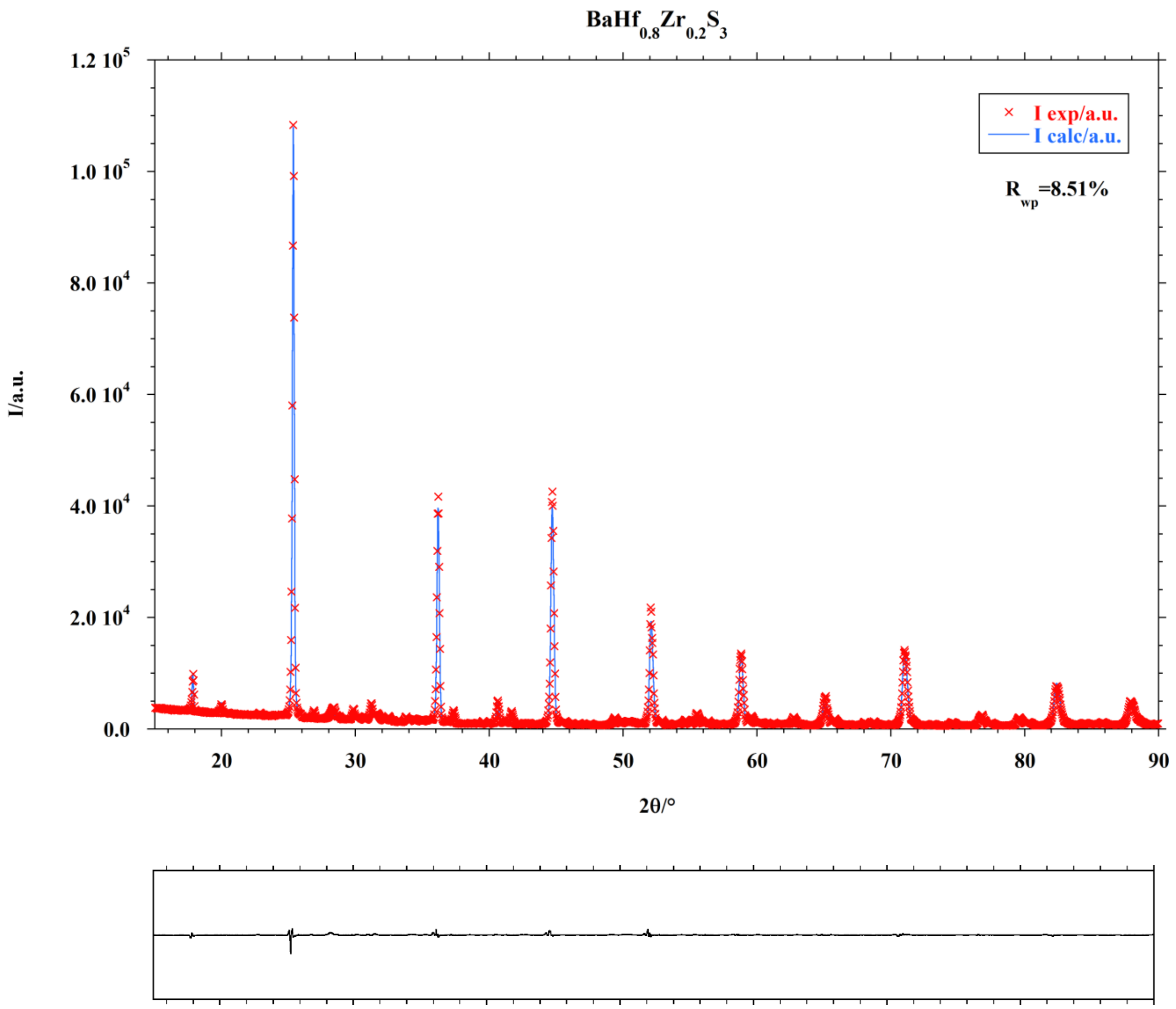

Figure S12. Experimental-calculated diffraction patterns of $BaHf_{0.8}Zr_{0.2}S_3$ (upper panel) and difference plot (lower panel).

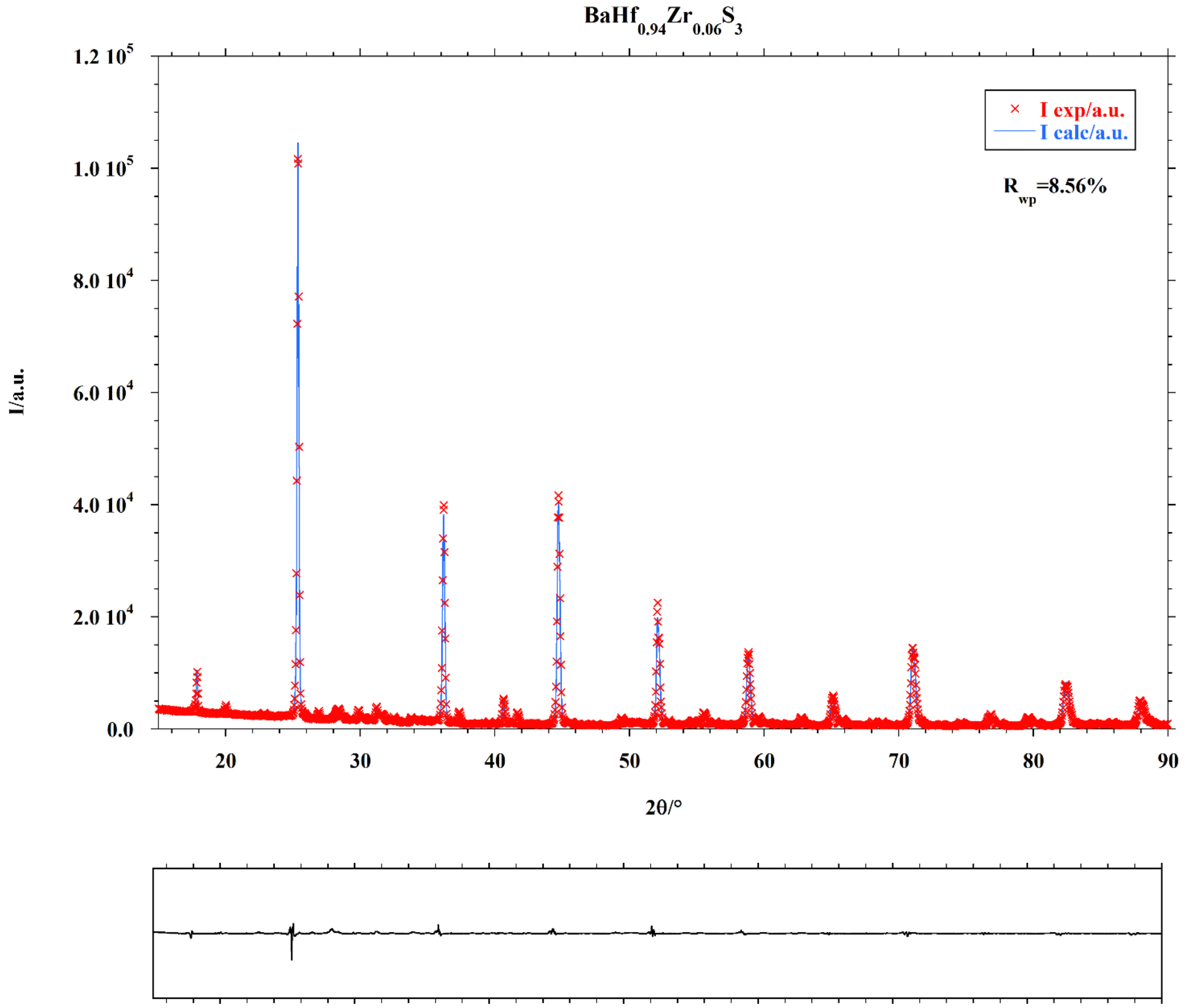

Figure S13. Experimental-calculated diffraction patterns of $BaHf_{0.94}Zr_{0.06}S_3$ (upper panel) and difference plot (lower panel).

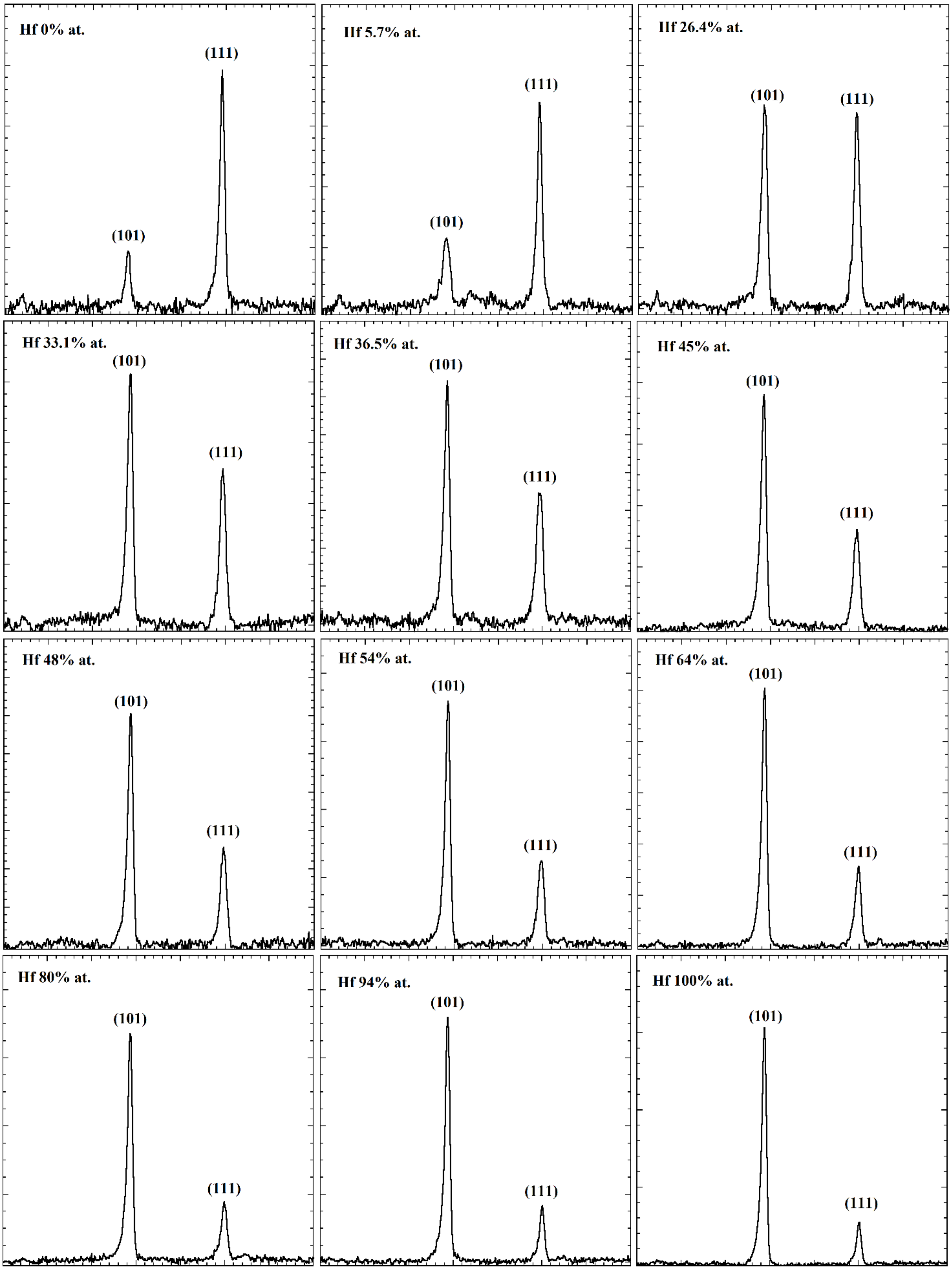

Figure S14. Relative variation of the (101) and (111) reflections intensities as a function of Hf content in some $BaHf_{(1-x)}Zr_xS_3$ solid solutions. In all graphs the abscissa range is 15-22° in 2θ.

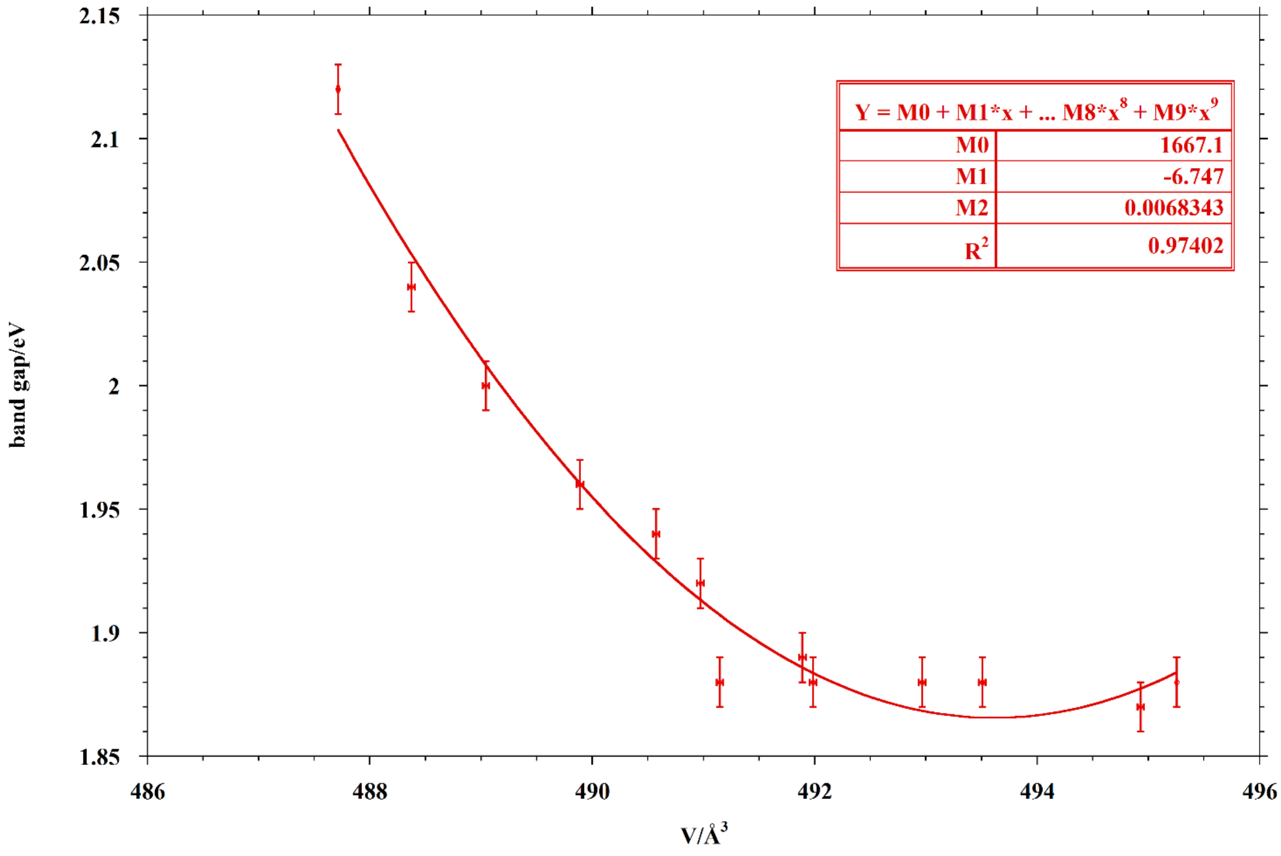

Figure S15. Band gap value vs. unit cell volume for $BaHf_{(1-x)}Zr_xS_3$ solid solutions.
